# Nanotwinning and structural phase transition in CdS quantum dots

**DOI:** 10.1186/1556-276X-7-584

**Published:** 2012-10-23

**Authors:** Pragati Kumar, Nupur Saxena, Ramesh Chandra, Vinay Gupta, Avinash Agarwal, Dinakar Kanjilal

**Affiliations:** 1 Department of Physics, Bareilly College, Bareilly, Uttar Pradesh, 243005, India; 2 Inter University Accelerator Centre, Aruna Asaf Ali Marg, P.O. Box 10502, New Delhi, 110067, India; 3 Institute Instrumentation Centre, Indian Institute of Technology, Roorkee, 247667, India; 4 Department of Physics & Astrophysics, Delhi University, Delhi, 110 007, India

**Keywords:** CdS quantum dots, CdS thin films, Pulsed laser deposition, Phase transition, Raman scattering, Photoluminescence, 78.67.Hc, 64.70.Nd, 81.07.Ta

## Abstract

Nanotwin structures are observed in high-resolution transmission electron microscopy studies of cubic phase CdS quantum dots in powder form by chemical co-precipitation method. The deposition of thin films of nanocrystalline CdS is carried out on silicon, glass, and TEM grids keeping the substrates at room temperature (RT) and 200°C by pulsed laser ablation. These films are then subjected to thermal annealing at different temperatures. Glancing angle X-ray diffraction results confirm structural phase transitions after thermal annealing of films deposited at RT and 200°C. The variation of average particle size and ratio of intensities in Raman peaks *I*_2LO_/*I*_1LO_ with annealing temperature are studied. It is found that electron-phonon interaction is a function of temperature and particle size and is independent of the structure. Besides Raman modes LO, 2LO and 3LO of CdS at approximately 302, 603, and 903 cm^−1^ respectively, two extra Raman modes at approximately 390 and 690 cm^−1^ are studied for the first time. The green and orange emissions observed in photoluminescence are correlated with phase transition.

## Background

Colloidal semiconductors' nanocrystals (NCs) have been rigorously studied by various researchers because their unique physical properties are a function of their particle size. The bandgap observed in the absorption and emission spectra of semiconductor quantum dots (QDs) are blueshifted due to confinement of charge carriers. The optical properties of nanoparticles can also be determined by coupling of confined charges and confined phonons. The physical properties, especially the light emitting properties, change drastically as the size of the semiconductor materials become smaller. The optical and electrical properties of semiconductor NCs for applications in optoelectronic devices [1,2] and biological fluorescence labeling [3,4] are affected by quantum confinement when their typical dimensions are equal to or smaller than the Bohr radius of exciton. Cadmium sulfide, with a bulk bandgap of approximately 2.42 eV (approximately 512 nm) and exciton Bohr radius of approximately 2.85 nm, is a candidate for quantum-dot blue light-emitting diodes [5]. It can also be used in photovoltaic devices. The microstructural characterization of NCs or QDs with size comparable to Bohr's exciton radius is useful to understand the light emission mechanism. Recently, our group reported the most common microstructural defects, i.e., twin structure, stacking faults, and grain boundaries in CdS QDs for the first time [6]. The surface and structural defects are expected to have important effects on the physical properties, particularly the optical properties of the QDs.

Thin films of wide bandgap II-VI semiconductors are of considerable interest as their emissions cover the technologically attractive blue and green spectral regions. In particular, CdS thin films attracted more attention because their bandgap emission is expected to lie very close to the highest sensitivity of the human eye, i.e., green light. The thin films of CdS quantum dots seek wide applications in photonic devices like laser, LEDs, etc. Ullrich and his group demonstrated optically pumped laser action in pulsed laser deposition (PLD)-grown CdS thin films [7,8]. Artemyev [9] and Nanda et al. [10] reported electroluminescence and photocurrent studies in devices fabricated using CdS nanocrystals. Nizamoglu et al. [11] fabricated white LEDs using CdS quantum dots hybridized on near-UV LEDs. Various methods have been used by different researchers to synthesize CdS nanocrystals thin films [12-17]. It has been shown that PLD is a versatile technique to maintain stoichiometry of film because of rapid temperature rise (>10^11^ K/s) produced by focused pulsed laser beam on the target [14]. Growth of high quality films at a relatively low substrate temperature by PLD is possible because high-energy atoms and ions in the laser-induced plasma plume create a higher surface mobility [18]. A lot of work has been reported on PLD grown CdS films investigating the effect of various parameters such as substrates, substrate temperature, laser fluence, laser wavelength, etc. [14,19-26]. Still, there is a need of further studies on PLD-grown films for the development of deeper understanding of their structures for future applications. To the best of our knowledge, the room temperature (RT) deposition of CdS thin film was never reported before. The thermal annealing-induced phase transition has been studied in thin films deposited by different routes [27-29], but not studied in PLD-grown films.

In this letter, we report on the studies of properties of CdS thin films grown by PLD, keeping the substrates at RT and 200°C, and the annealing effects on the structural and optical properties of the films. The variation of average particle size and ratio of intensities of Raman peaks *I*_2LO_/*I*_1LO_ are studied with respect to the annealing temperature. It seems that electron-phonon interaction is a function of temperature and particle size, irrespective of the structure. Two extra modes in Raman spectra have been identified for the first time. These are verified by low frequency Raman studies.

## Methods

Thin films of CdS quantum dots are deposited by laser ablation of a target prepared by pressing and sintering the chemically synthesized CdS QDs powder. The synthesis of CdS quantum dots in powder form is reported elsewhere [6]. For PLD, using ultraviolet laser source, a pulsed excimer KrF laser (Lambda Physik, Compex Pro 201, Coherent Inc., CA, USA) operating at 248-nm wavelength has been used. The pulse width of 10 ns and energy of 300 mJ per pulse have been used. The laser beam with a repetition rate 10 Hz is focused onto a rotating target mounted at an oblique angle of 30°. The distance between target and substrate is kept as 5.5 cm. The films are deposited on single-crystal (111) n-type silicon wafers, glass and carbon-coated Cu grids (for TEM) at two different temperatures: (1) RT and (2) 200°C inside a clean stainless steel vacuum chamber with a background pressure of 5 × 10^−6^ mbar. The silicon and glass substrates are cleaned using a standard process that involves boiling in trichloroethylene followed by rinsing with deionized water. The CdS deposition rate in this configuration is about 0.025 nm per pulse. The thickness of deposited film is about 250 nm. To study the post-annealing effect on structural and optical properties of the films, thermal annealing is carried out at different temperatures 300°C to 450°C for 3 h in Ar environment.

Glancing angle X-ray diffraction (GAXRD) studies are carried out at an angle of 1° using Bruker D8 diffractometer (Bruker AXS GmbH, Germany; Cu K_α_ radiation, *λ* = 1.5406 Å) and micro-Raman spectroscopy using Renishaw Invia Raman microscope (Renishaw plc, Gloucestershire, UK) with 514-nm excitation wavelength of an Ar ion laser. The low frequency micro-Raman scattering measurements were performed in the backscattering geometry using a Jobin Yvon T64000 triple monochromator (NJ, USA) with a Coherent INNOVA 99 Ar^+^ laser (514.5 nm) equipped with a charge-coupled device detector. The samples are examined by transmission electron microscopy (TEM) and high resolution transmission electron microscopy (HRTEM) using Tecnai G20-Stwin operating (FEI Company, Shanghai, China) at 200 kV with point resolution of 1.44 Å, line resolution of 2.32 Å, and line-type super twin lenses. The films deposited on glass substrates are analyzed using UV-vis absorption spectroscopy (Hitachi 3300 UV/visible spectrophotometer; Hitachi High-Technologies Corporation, Tokyo, Japan). Photoluminescence (PL) spectroscopy studies are carried out at room temperature using HORIBA Jobin Yvon LabRAM 800 HR (NJ, USA) with excitation wavelength at 325nm from He-Cd laser.

## Results and discussion

**Figure 1:**
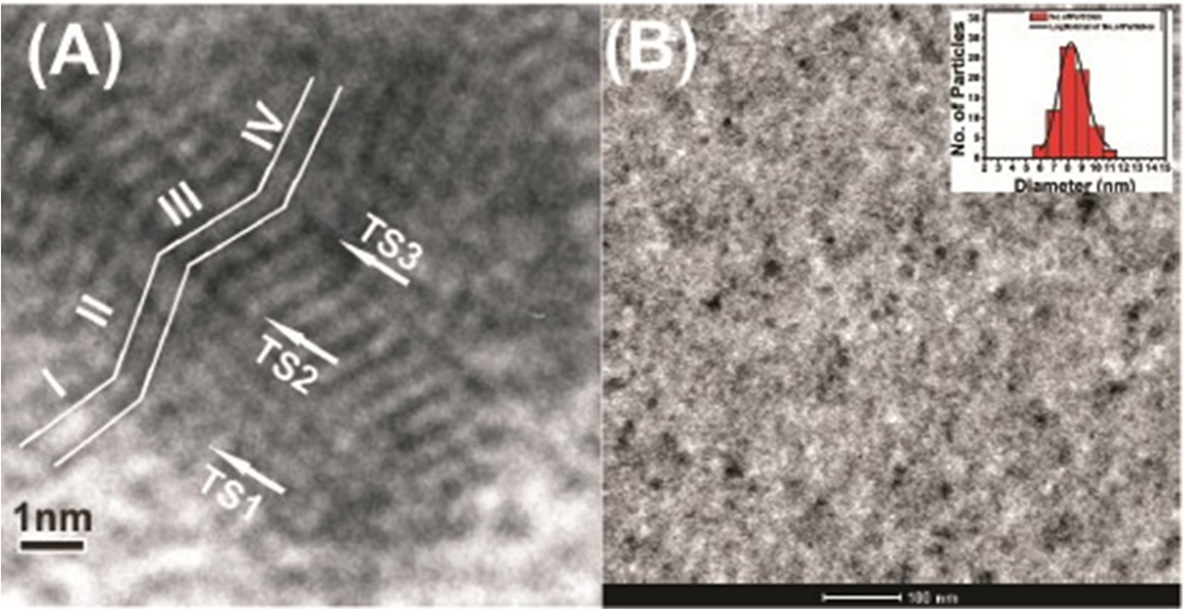
**HRTEM and TEM micrographs of CdS QDs.** (**a**) HRTEM micrograph of CdS QDs and (**b**) TEM micrograph of CdS film grown at RT and particle size distribution (inset).

CdS quantum dots in powder form are characterized using high resolution transmission electron microscopy to study the microstructure and defects present in the dots. The existence of multi-twin structure can be clearly seen in Figure 1a. The twin structures exist with stacking fault and grain boundaries in chemically synthesized CdS QDs of average particle size of approximately 2.7 nm [6]. CdS thin films are deposited by PLD using a target prepared by these CdS quantum dots. TEM micrograph of CdS thin film grown at RT is shown in Figure 1b. It exhibits uniformly distributed CdS nanoparticles of average size of approximately 8.3 nm with a narrow size distribution, as shown in the inset of Figure 1b.

**Figure 2:**
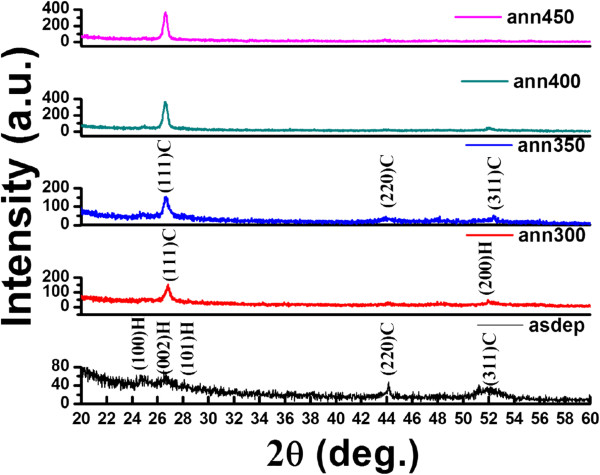
GAXRD pattern of CdS thin films deposited at RT and annealed at different temperatures.

**Figure 3:**
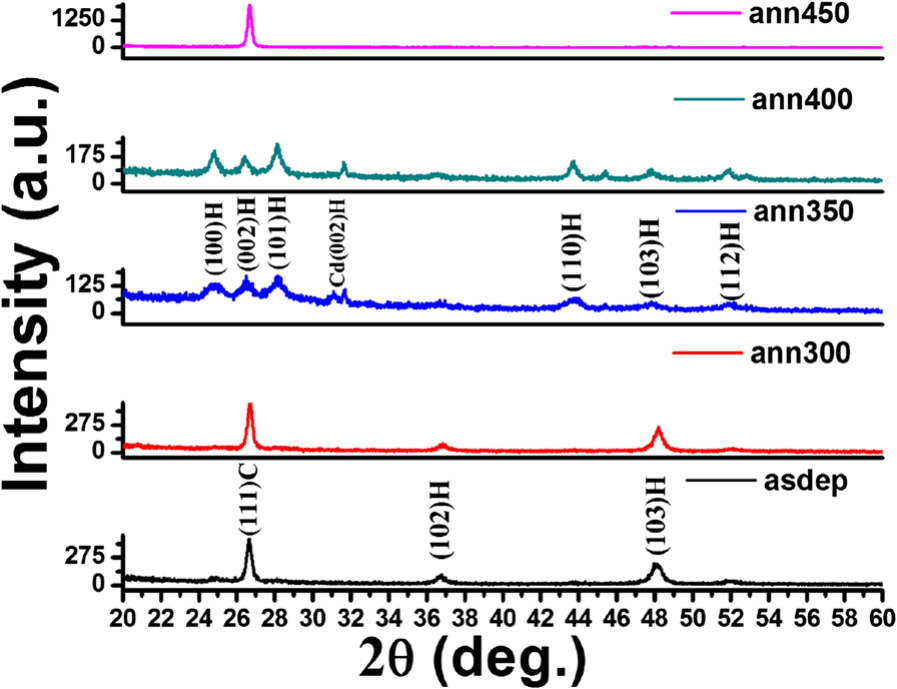
GAXRD pattern of CdS thin films deposited at substrate temperature 200°C and annealed at different temperatures.

Results of GAXRD scan of films deposited at two different temperatures, (1) RT and (2) 200°C, and further annealed at different temperatures in Ar environment are given in Figures 2 and 3, respectively. From Figure 2, it is seen that as-grown film at RT is in mixed phase of cubic and hexagonal structures. The diffraction peaks at 24.67°, 26.60°, and 28.1° correspond to (100), (002) and (101) planes of the hexagonal structure. The peaks at 44.15° and 52° are assigned to (220) and (311) planes of cubic structure. It is observed that after annealing, diffraction peaks at 24.67° and 28.1° disappear, whereas the intensity of the peak at 26.60° is enhanced. It is seen from Figure 2 that the mixed phase of as-deposited film transferred to cubic phase after annealing at 350°C as confirmed by the appearance of diffraction peaks at 26.60°, 43.9°, and 52.25° corresponding to (111), (220), and (311) planes (PCPDF WIN 100454) of the cubic phase. Further annealing at higher temperature enhances the intensity of peak at 26.6° corresponding to (111) plane of the cubic phase, and the intensity of other peaks is reduced. Increase in intensity and sharpness of diffraction peak corresponding to the plane (111) with increasing annealing temperature shows that the crystallinity of the film is improved.

Figure 3 shows that as-grown film at substrate temperature of 200°C is also in mixed phase of cubic and hexagonal structures but with different orientation of planes. The diffraction peak at 26.64° is assigned to (111) plane of the cubic structure, whereas the peaks at 36.8° and 48.1° are assigned to (102) and (103) planes of the hexagonal structure, respectively. It is clearly seen from Figure 3 that comparatively stable hexagonal phase starts to dominate over the mixed phase at annealing temperature of 350°C. Complete hexagonal phase occurs at 400°C. The diffraction peaks at 24.8°, 26.48°, 28.12°, 43.64°, 47.8°, and 51.9° correspond to the (100), (002), (101), (110), (103), and (112) planes (PCPDF WIN 751545) of the hexagonal structure with lattice parameters *a* = 4.12 Å and *c* = 6.724 Å for the film annealed at 350°C. There is one additional diffraction peak at 31.6° corresponding to (002) plane of the hexagonal phase of Cd. It is also clear from Figure 3 that there is a phase transition from hexagonal to cubic phase for the film annealed at 450°C.

Cadmium sulfide has C_6V_ symmetry with four atoms per unit cell. Group theory predicts that there are nine optical branches at the zone center. These optical branches can be classified as one symmetric A_1_ and one doubly degenerate E_1_ which are both Raman and infrared active, two doubly degenerate E_2_ branches which are Raman active only, and two antisymmetric with respect to the twofold and sixfold axes (B_1_) ‘silent modes’ inactive in both infrared absorption and Raman scattering [30,31].

**Figure 4:**
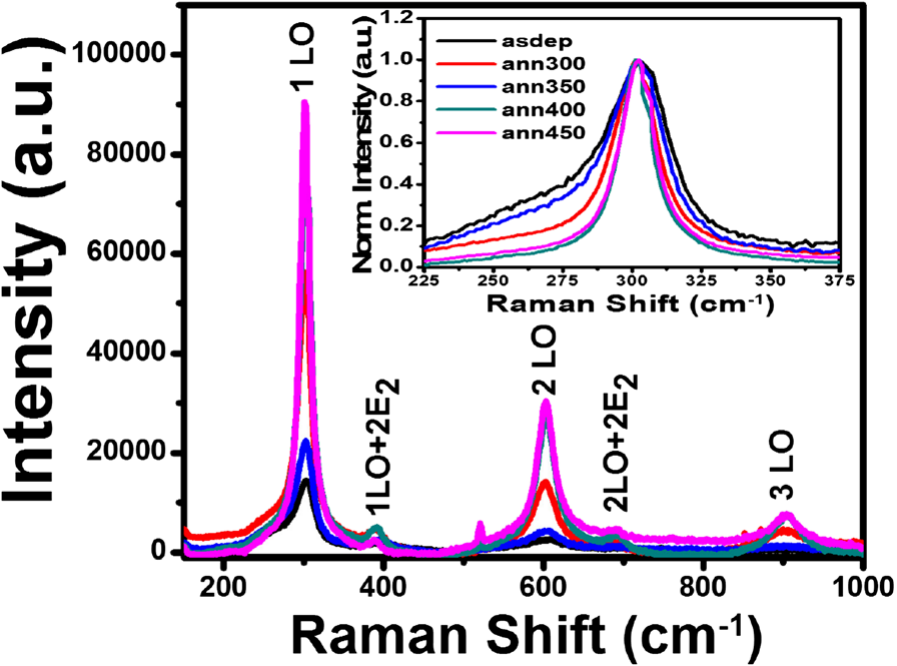
**Raman spectra of CdS thin films deposited at RT and annealed at different temperatures.** Inset image is the normalized intensity vs. Raman shift of 1LO mode.

**Figure 5:**
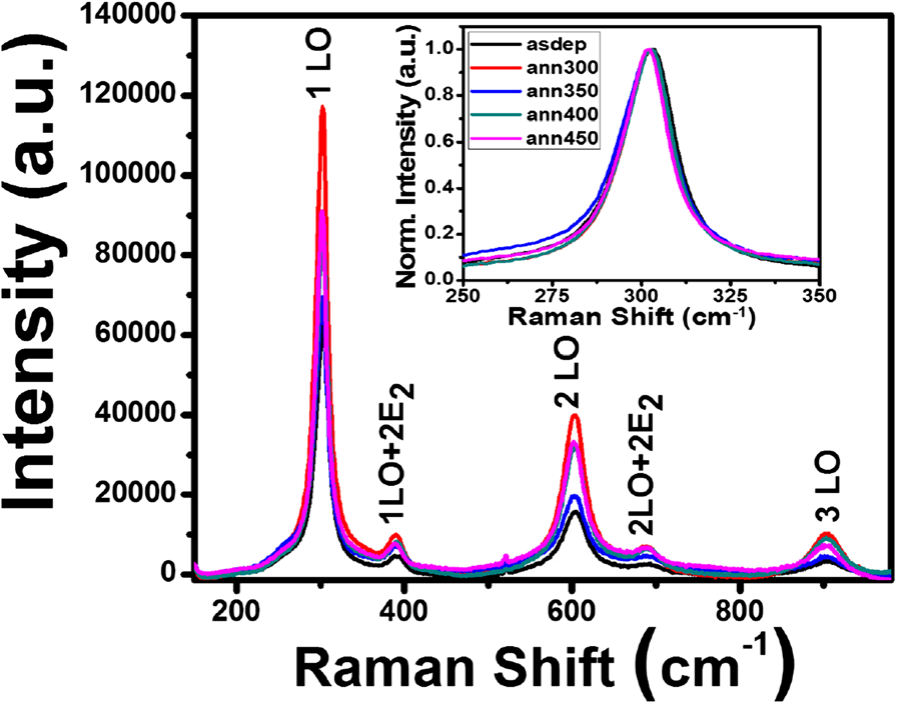
**Raman spectra of CdS thin films deposited at 200°C and annealed at different temperatures.** Inset image is the normalized intensity vs. Raman shift of 1LO mode.

**Figure 6:**
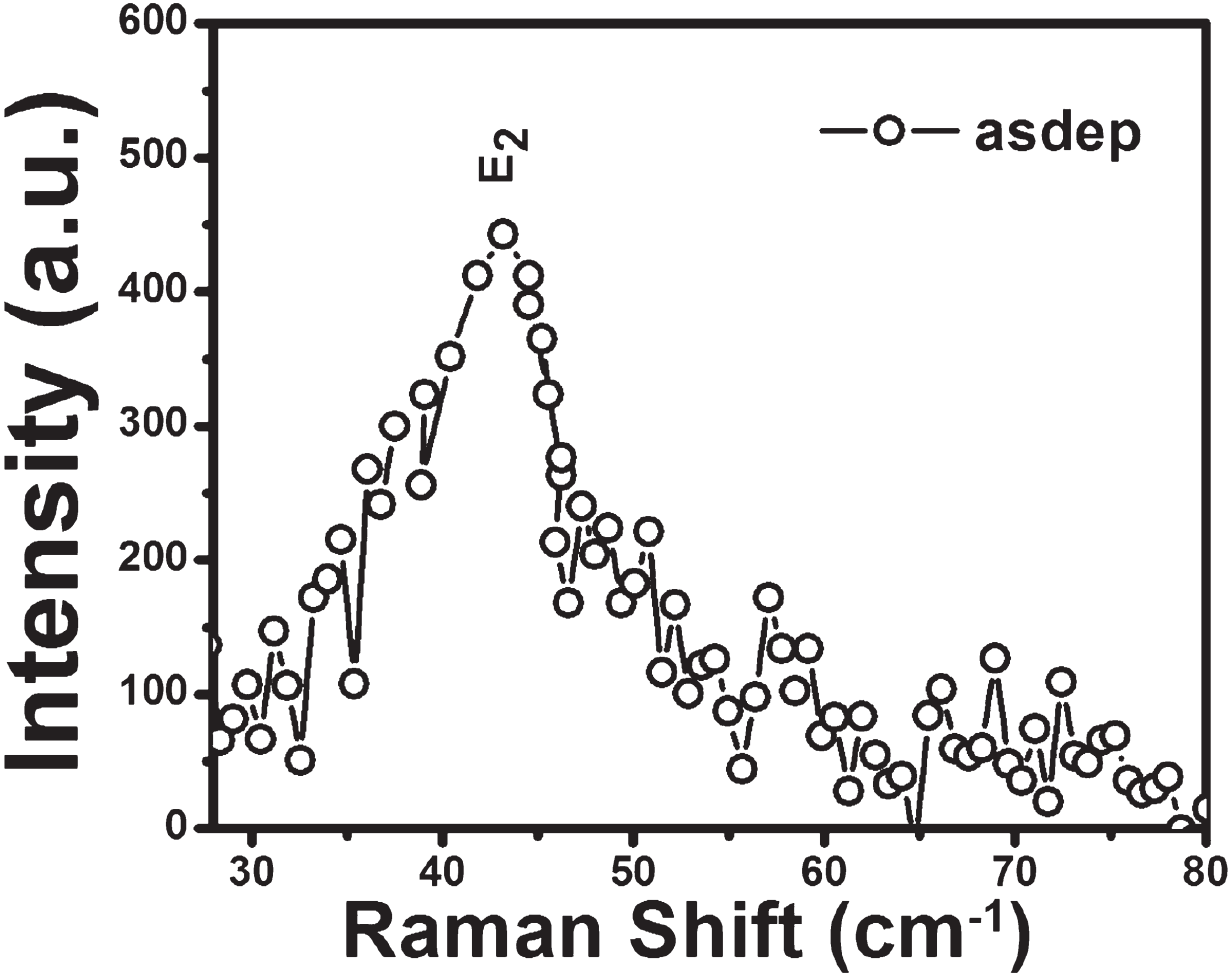
Low frequency Raman spectrum of CdS thin film deposited at substrate temperature 200°C.

In bulk CdS crystals, the phonon eigenstate is a plane wave, and the selection rule for Raman scattering is ***q*** ≈ 0, where ***q*** is the wave vector. However, in nanocrystalline materials, ***q*** ≈ 0 selection rule is relaxed due to interruption of lattice periodicity. In the present study, we observe five optical vibrational Raman active modes at approximately 302, 390, 603, 690, and 903 cm^−1^ in all samples as shown in Figures 4 and 5. The intense and broad peaks at approximately 302, 603, and 903 cm^−1^ are assigned to fundamental optical phonon mode (LO), the first over tone mode (2LO), and the second overtone (3LO) of CdS, respectively. These are in agreement with previous reports [32]. In nanometer-sized particles, the most prominent peak of CdS may be shifted to 300 cm^−1^[32]. The reduction in particle size causes noticeable asymmetry and frequency shift towards the lower frequency side, which has been theoretically calculated and experimentally observed [33]. Raman spectra in Figures 4 and 5 show that the first-order LO Raman line is not only broadened but also asymmetric towards the lower frequency side compared to bulk CdS (305 cm^−1^). The weak Raman peaks at approximately 390 and 690 cm^−1^ result from multiphonon scattering. They are identified as those corresponding to the vibrational modes 1LO + 2E_2_ and 2LO + 2E_2_ respectively. The peak at 394 cm^−1^ is observed previously for CdS microcrystallite-doped glass thin film by Jerominek et al., but it remained unidentified [34]. To confirm our observation related to these modes, low frequency Raman spectrum of as-deposited CdS film deposited at 200°C is shown in Figure 6. Low frequency Raman spectrum shows the presence of E_2_ branch at approximately 43.3 cm^−1^ which is reported at 44 cm^−1^ for bulk CdS in the literature [31].

**Figure 7:**
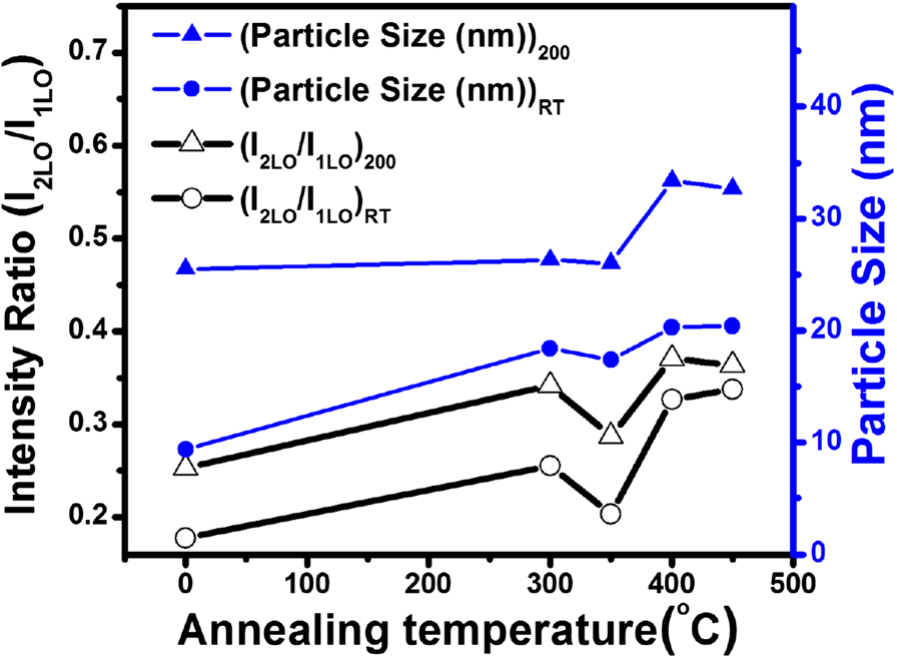
**Particle size and*I***_**2LO**_**/*I***_**1LO**_**variation with annealing temperature for films grown at RT and 200°C.***I*_2LO_ and *I*_1LO_ represent intensity of 2LO and 1LO Raman modes, respectively.

It can be clearly seen from Figures 4 and 5 that the peak intensities of 1LO, 2LO, and 3LO modes increase first for films annealed at 300°C, suddenly decrease for films annealed at 350°C, and then continuously increase up to highest annealing temperature. According to Sivasubramanian et al., this is due to the phase transition because change in structure may be the factor, which results in the change in the electron-phonon coupling [35]. As the particle size increases with annealing, 2LO line becomes stronger, while the 1LO line becomes weaker in intensity. The variation of intensity ratio *I*_2LO_/*I*_1LO_ as a function of annealing temperature is shown in Figure 7. The average particle size is calculated for all samples from GAXRD analysis using Debye Scherrer's formula, and its variation is plotted with annealing temperature in Figure 7. It can be seen that the behavior of Raman intensities of the fundamental and its overtone for mixed, cubic, and hexagonal phases are different. However, in either of the phases, the ratio *I*_2LO_/*I*_1LO_ increases as the temperature/particle size increases. Since the strength of the electron-phonon interaction is measured by *I*_2LO_/*I*_1LO_ ratio [35], it may be concluded from the present study that electron-phonon interaction is a function of temperature and particle size, irrespective of the structure. In bulk CdS, there is comparatively strong electron-phonon coupling in hexagonal phase than that in the cubic phase [36]. It may be concluded that electron-phonon coupling in mixed phase is comparatively weaker than that in the hexagonal or cubic phase. This is because of the difference in the symmetries of LO phonons together with nature of the interband transitions in either of the two phases. Asymmetry of 1LO mode (Figure 4 and Figure 5 inset) varies with electron-phonon interaction, annealing temperature, and particle size. The intensity of 1LO + 2E_2_ and 2LO + 2E_2_ decreases as annealing temperature is increased. This is in agreement with previous result of Jerominek et al. [34]. They observed that peak at 394 cm^−1^ vanished in film annealed at 500°C.

**Figure 8:**
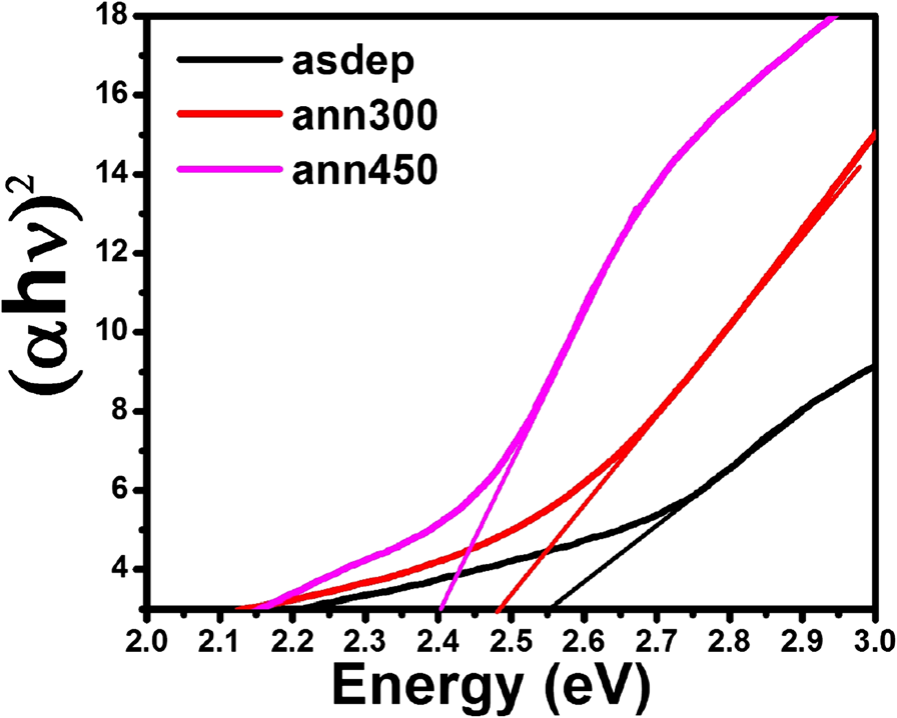
Tauc plot of CdS thin films deposited at RT and annealed at different temperatures.

**Figure 9:**
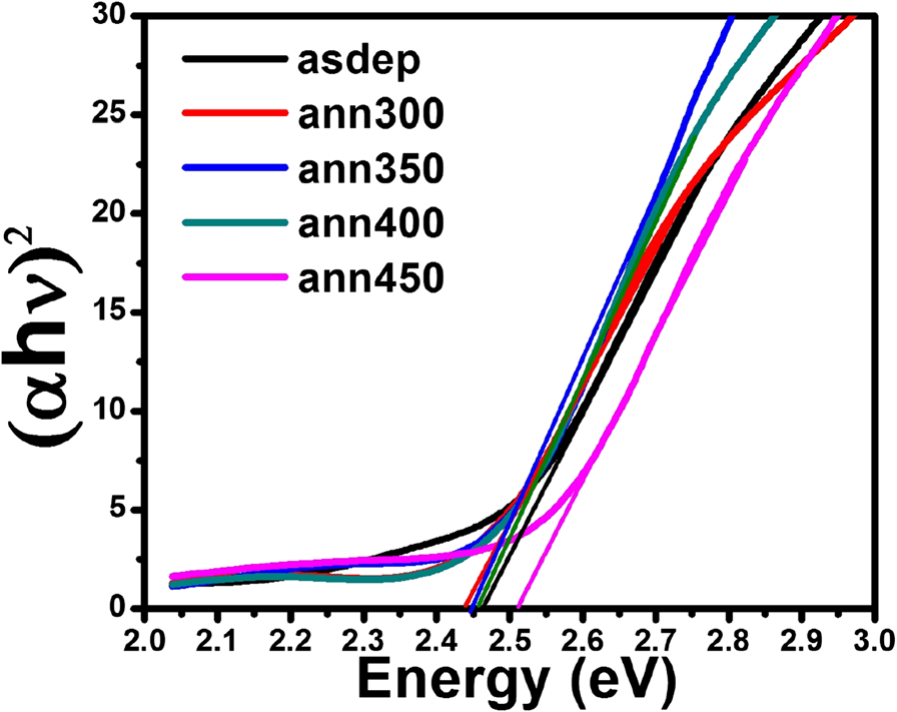
Tauc plot of CdS thin films deposited at 200°C and annealed at different temperatures.

UV-visible absorption spectra are recorded for the films deposited on glass substrate to study the annealing effect on energy bandgap of CdS thin films. Figures 8 and 9 show the Tauc plots of the films grown at RT and 200°C, and annealed at different temperatures. The direct bandgap values are calculated as approximately 2.55, 2.39, and 2.48 eV for as-deposited, annealed at 300°C, and 450°C, respectively, for films grown at RT by extrapolating the linear fit to the energy axis from the plot of (*αhν*)^2^ versus energy. The bandgap of as-grown film at 200°C is estimated as approximately 2.46 eV and becomes minimum for film annealed at 300°C. Further annealing results in increase in bandgap. It becomes nearly equal to the bandgap of as-deposited film (approximately 2.44 eV) for the film annealed at 400°C. These results are in agreement with previous report [36]. It can be seen from Figure 9 that at 450°C, the bandgap increases suddenly. This may be due to the unexpected phase transition from hexagonal to cubic structure at 450°C.

**Figure 10:**
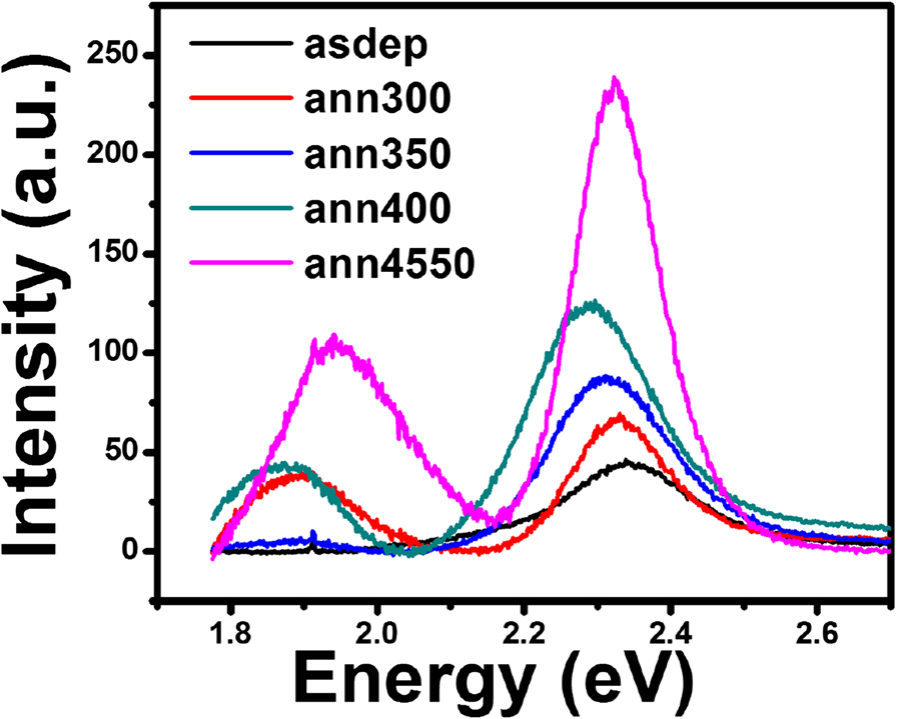
PL spectra of CdS thin films deposited at substrate temperature 200°C and annealed at different temperatures.

Photoluminescence studies are carried out to get the information regarding different energy states available between valence and conduction bands, which are responsible for radiative recombination. PL spectra for the films deposited at 200°C and annealed at different temperatures are shown in Figure 10. It reveals that as-deposited film shows broad green emission centered at approximately 2.35 eV. The green emission in as-deposited film may be attributed to the transition of S-vacancy donors to the valence band. It is expected that during deposition at substrate temperature 200°C, some sulfur vacancies may be created. The intensity of green emission increases with the increase in annealing temperature and shifts towards lower energy side from 2.35 to 2.28 eV. It seems that the increase in intensity and shifting toward lower energy side is due to the increase in sulfur vacancies with rise in temperature. It can also be seen from Figure 10 that annealing results in an additional broad orange emission peak at approximately 1.87 eV whose intensity increases with rise in temperature except for the film annealed at 350°C where intensity is decreased drastically. The origin of the orange emission may be due to the transition between interstitial cadmium donor level and acceptor level located at higher energies with respect to the valence band [36]. The decrease in intensity of this peak at 350°C may be due to the structural phase transition.

## Conclusions

The influence of annealing in Ar environment on PLD-grown CdS thin films at two different temperatures, (1) RT and (2) 200°C has been studied. The annealing-induced phase transition is observed in both types of films with different features. The structural phase transition is correlated with variation in particle size, bandgap, intensity ratio of 2LO to 1LO (*I*_2LO_/*I*_1LO_) Raman peaks, asymmetry in 1LO mode, and PL results. It may be concluded that in either of the phases *viz.* mixed, cubic or hexagonal, the ratio *I*_2LO_/*I*_1LO_ increases as the temperature and particle size increase, and electron-phonon interaction is a function of temperature and particle size, irrespective of the structure. The existence of the two extra Raman active modes at approximately 390 and 690 cm^−1^ is observed using low frequency Raman measurement.

## Competing interests

The authors declare that they have no competing interests.

## Authors’ contributions

PK conceived the idea. PK and NS performed the experiments. AA and DK supervised the project. RC and VG provided the facilities and discussions related to them. PK and NS co-wrote the paper. All the authors read and approved the final manuscript.

## Authors’ information

PK is a research scholar under the supervision of AA, an assistant professor at the Department of Physics, Bareilly College, Bareilly, India. NS is research associate working with DK at Inter University Accelerator Centre, New Delhi, India. RC and VG are professors at Indian Institute of Technology, Roorkee and University of Delhi, respectively.
